# News and Notes

**Published:** 1906-09

**Authors:** 


### NEWS AND NOTES.

A MARINE hospital is to be erected in Pittsburg, in the grounds of the Allegheny Arsenal.

THE College of Physicians and Surgeons has been recently organized in Little Rock, Ark.

THE subscriptions to the relief fund of San Francisco's great disaster amount to $7,135,305.89.

TWENTY-FOUR of the 511 Seniors at Harvard University this year have decided to study medicine.

THE annual session of the British Medical Association was held this year in Toronto, August 21-25.

PROFESSOR ALMWORTH EDWARD WRIGHT has been knighted by King Edward in recognition of his discovery of the opsonic index.

OKLAHOMA and Indian Territory have shown the largest percentage increase in the number of physicians during the past five years.

AT the recent session of Congress, $75,000 was appropriated for the construction of an assembly hall at the Government Hospital for the Insane.

THE epidemic of typhoid fever in Washington, D. C., is becoming quite serious, as the number of cases is increasing in a greater ratio than last year.

THE Ceylon government has established a pathologic laboratory in Colombo, under the direction of Dr. Castellani, for the study of tropical medicine.

---

THE new Virchow Hospital of Berlin is to have 800 beds. Its total cost will reach five million dollars, and the attending physicians will have annual salaries.

---

THE Association of Surgeons of the Seaboard Air Line Railroad held its annual meeting at Savannah, Georgia, on Friday and Saturday, July 13th and 14th.

---

A TAX on prescriptions has been proposed in Austria to obtain funds for improvement of the institution for the orphans and widows of medical practitioners.

---

MANUEL GARCIA, inventor of the laryngoscope, and the most famous vocal teacher of his time, died in London, July 2, 1906, in his one hundred and first year.

---

HENRIK IBSEN, the great Norwegian dramatist and poet, who died last month, began his business career as apprentice in a small apothecary shop in a Norwegian town.

---

DR. S. A. WILSON died August 5th at his home in Battle Hill, Ga., after an illness of three weeks, from pneumonia. Dr. Wilson is survived by his wife and one son.

---

FLORAL POLLOCK, M.D., reports that of the 1,089 patients treated in the Women's Venereal Department of Johns Hopkins Hospital Dispensary, more than sixty per cent. were wives and children, and nearly all ignorant victims of these diseases, only a very small per cent. being prostitutes.

DR. J. B. S. HOLMES has moved from Atlanta to Valdosta, Ga., where he has opened up an up-to-date sanatorium. The many friends of Dr. Holmes in Atlanta wish him success.

---

DR. JAMES B. MCCAW, of Richmond, Va., died August 13th, aged eighty-four years. Dr. McCaw was a prominent surgeon in the Confederate Army during the war between the States.

---

ON account of the numerous arrests and banishments of medical men, and of the boycotting of certain localities by sympathetic physicians, there is a great scarcity of medical attendance in many sections of Russia.

---

A MERGER of the Chicago Medical College and the Northwestern University was consummated July 24th, when the Chicago Medical College gave a quitclaim deed to the Northwestern University for all its property.

---

A house-boat has been prepared to be used at the mouth of the Mississippi to isolate crews from infected ports, and to enable fruit steamers to carry their perishable cargoes in charge of other crews through New Orleans, thereby avoiding delay.

---

ASSISTANT Surgeon Harry L. Brown, a native of Maryland, and Assistant Surgeon Theodore U. Pease, of Massachusetts, are to be tried at the navy yard in Washington by court-martial on the charge of having cheated in their examinations for promotion.

---

IN spite of the safeguards thrown around the milk supply of New York, the high mortality among infants has led Nathan Straus to advance the claim that the only remedy is to pasteurize all the milk brought into the city, as a large number of these deaths are still due to infected milk.

THE May-June issue of the *Pacific Medical Journal* is dedicated to the members of the medical profession of San Francisco "for the noble, self-sacrificing and heroic work done by them during the greatest calamity that has ever befallen a stricken people."

---

THE census bureau reports that the annual cost of maintenance of the insane in public hospitals is approximately $21,000,000, not including the income from pay patients and other sources. According to this report, insanity is increasing in the United States.

---

A gift of $5,000 has been made to the New York Postgraduate Medical School and Hospital, by Mr. and Mrs. Edgar E. Brandon, of Oxford, Ohio, "in memory of their infant son, William Templeton Brandon," to establish an obstetrical ward in connection with the hospital.

---

DR. C. P. THOMAS, of Spokane, probably holds the record for automobile night runs over unfamiliar territory, having recently covered ninety-six miles in three hours and a half to a lumber-camp north of Spokane. The auto was pressed into service as there was no train for the north country until the following morning.

---

ACCORDING to Henry Hun, M.D., the real origin of the trained nurse was in the convents of Asia Minor, in the Holy Land. From here the convents spread westward over Europe. During the crusades many "Hospices" were established for the care and nursing of the pilgrims and crusaders who fell ill on their journey, although this was not their only duty.

---

MR. ARNOLD LUPTON, a *sagacious solon* and member of the British Parliament, has recently made the ludicrous statement that

100,000 people are killed every year in England alone by vaccination. He has arrived at these figures by showing that the mortality from fifteen various diseases has increased since the introduction of compulsory vaccination. The increase in population and many other pertinent factors which have without doubt an influence over the mortality records have naturally been overlooked by this astute ( ?) and over-zealous statesman.

---

### TRI-STATE (ALABAMA, GEORGIA, TENNESSEE) MEDICAL SOCIETY.

The eighteenth annual meeting of the Tri-State Medical Society of Alabama, Georgia and Tennessee, will be held at Chattanooga, October 2-4, 1906. Reduced rates have been obtained from all points in Alabama, Georgia, Tennessee, Mississippi, Louisiana and Florida, and an unusually large attendance is assured.

The preliminary program includes an excellent list of papers from leading medical men of the South. Strong pressure will be brought to bear to ultimately convert this organization into a branch of the A. M. A.—The Association of the Southeastern States—and recommendations will be made at this meeting.

Physicians desiring to read papers should send their titles at once to the secretary, Dr. Raymond Wallace, Chattanooga, Tennessee.

---

FRACTURE of the outer end of the clavicle may follow a fall upon the shoulder. Unless one makes a careful examination such a fracture may escape observation or be mistaken for a dislocation of the outer end of the clavicle, with which condition, indeed, it may be associated.—*American Journal of Surgery.*

